# Enhanced Immunogenicity of Foot-and-Mouth Disease Virus-like Particles Using a Water-in-Oil-in-Water Adjuvant

**DOI:** 10.3390/vaccines13010024

**Published:** 2024-12-30

**Authors:** Yujie Zhou, Wenzhu Yin, Zhidong Teng, Yanyan Zhao, Yu Lu, Yingjuan Qian, Bihua Deng

**Affiliations:** 1 MOE Joint International Research Laboratory of Animal Health and Food Safety, College of Veterinary Medicine, Nanjing Agricultural University, Nanjing 210095, China; 2 Institute of Veterinary Immunology & Engineering, Jiangsu Academy of Agricultural Sciences, Nanjing 210014, China; 3 College of Veterinary Medicine, Nanjing Agricultural University, Nanjing 210095, China; 4 GuoTai (Taizhou) Center of Technology Innovation for Veterinary Biologicals, Taizhou 225300, China; 5 State Key Laboratory of Veterinary Etiological Biology, National Foot-and-Mouth Disease Reference Laboratory, Lanzhou Veterinary Research Institute, Chinese Academy of Agricultural Sciences, Lanzhou 730046, China; 6 Public R&D Platform of Veterinary Vaccines Molecular Design and Formulation Ministry of Agriculture and Rural Affairs, Nanjing 210031, China

**Keywords:** foot-and-mouth disease, virus-like particle, W/O/W double emulsion, adjuvant

## Abstract

Background: Foot-and-mouth disease (FMD) causes significant economic losses, prompting vaccination as a primary control strategy. Virus-like particles (VLPs) have emerged as promising candidates for FMD vaccines but require adjuvants to enhance their immunogenicity. In this study, we evaluated the immunogenicity of a VLP-based vaccine with a water-in-oil-in-water (W/O/W) emulsion adjuvant, named WT. Methods: The WT adjuvant was mixed with FMD VLPs to form the VLPs+WT vaccine. The size and stability of the vaccine were analyzed. BALB/c mice were immunized with the VLPs+WT vaccine, and immunological responses were assessed through antibody measurements, cytokine profiling, and gene expression analysis. In addition, splenic lymphocyte proliferation and signaling pathways were examined. Results: The VLPs+WT vaccine exhibited a homogeneous size of 324.60 ± 2.30 nm and a viscosity of 8.76 mPa·s, indicating good stability. Immunized mice showed steady weight gain and no organ abnormalities. Compared to the VLPs group, the VLPs+WT group induced significantly higher levels of specific antibodies that persisted for 12 weeks, similar to the commercial VLPs+ISA201 vaccine. The VLPs+WT vaccine also enhanced the secretion of Th1-related (IgG2a, IFN-γ) and Th2-related (IgG1, IL-4) molecules. WT stimulated splenic lymphocyte proliferation and differentiation, primarily activating B-cell receptor signaling and phagosome pathways. It also upregulated genes associated with MHC and interferon stimulation while promoting the expression of MyD88, PI3K, AKT, p65, and p-p65 proteins. Conclusions: These findings suggest that WT is an effective adjuvant for FMD VLP-based vaccines, with potential for improving vaccine efficacy.

## 1. Introduction

Foot-and-mouth disease (FMD) is a highly contagious disease caused by the FMD virus. It primarily infects cloven-hoofed animals such as pigs, cattle, and sheep, leading to weight loss, reduced milk production, and stunted growth in infected animals [1]. The disease can also severely restrict national and international trade, causing significant economic losses to the livestock industry worldwide [2,3,4,5]. At present, there is no effective treatment for FMD-infected livestock, and vaccination is the best option for preventing and controlling the disease. Although inactivated FMD vaccines have made significant contributions, they present challenges such as the need for high-level biosafety facilities, potential risk of virus escape, and difficulties in culturing some new serotypes or subtypes on cells [1,6,7].

**Figure 1:**
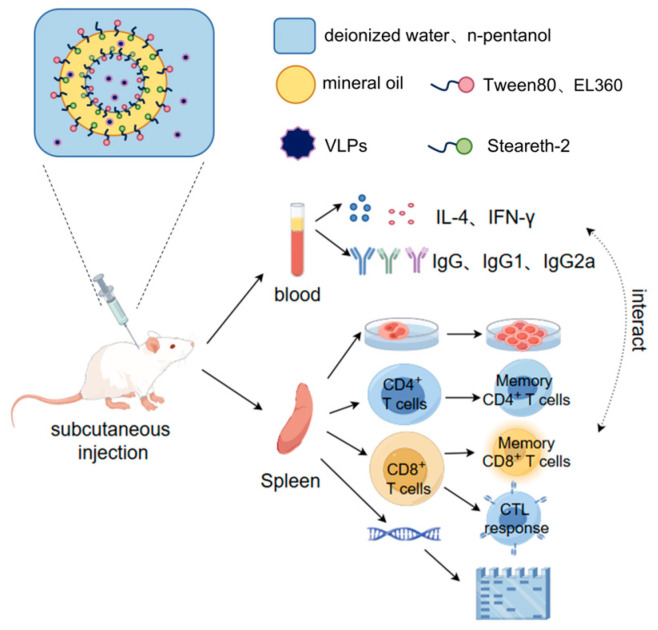
Schematic diagram of the experimental procedure, including preparation, lymphocyte proliferation in vitro, and a series of immune responses in vivo.

To overcome these issues, new types of vaccines are gradually becoming the trend [8,9,10,11]. Virus-like particle (VLP) vaccines have been proven to have reliable immunogenicity and safety in preclinical and clinical studies [12,13]. However, FMD VLPs alone cannot elicit sufficient immune responses and still require adjuvants to induce a higher immune response. Currently, adjuvants added to FMD VLPs include chitosan [11], liposomes [14], gold nanoparticles [15,16], saponins [11], oil-based emulsions [17,18], etc. Among these, FMD VLP vaccines with chitosan effectively activate mast cells in lymph nodes but do not significantly promote IgG levels and lymphocyte proliferation. Liposome adjuvants cannot encapsulate large viruses. Although gold nanoparticles have been shown to have low toxicity, they still need to pay attention to metabolic issues. Oil-based emulsion adjuvants can save antigen doses, slowly release antigens, enhance adaptive immune responses [19], have good stability, and be stored for a long time [20]. Compared to traditional alum or saponin vaccines, oil-based vaccine formulations exhibit stronger and more durable immune responses and earlier protective effects [21]. The most widely used FMD vaccine adjuvants on the market are ISA201 and ISA206 [22,23,24], both of which are low-viscosity water-in-oil-in-water (W/O/W) emulsions [25,26]. This study aims to evaluate the efficacy of our previously developed W/O/W adjuvant, referred to as WT adjuvant, in FMD VLP vaccines. Prior research has demonstrated that the WT adjuvant enhances immune response in inactivated FMD vaccines [27]. As shown in Figure 1, this study will evaluate the physicochemical properties, safety, immunogenicity, and related mechanisms of the VLPs+WT vaccine, which may help inform future strategies for FMD vaccine development.

## 2. Materials and Methods

### 2.1. Materials

FMD VLPs were provided by professor Huichen Guo (Lanzhou, China), and the VLP samples exhibited an approximate diameter of 25 nm [28]. Mineral oil HND-1 was purchased from CNOOC Petrochemical Co., Ltd. (Taizhou, China). EL360, Tween-80, n-pentanol, and steareth-2 were all of analytical reagent grade and were purchased from Sinopharm Chemical Reagent Co., Ltd. (Shanghai, China). ISA201 was sourced from Seppic (Puteaux, France). The FMD O-type antibody liquid-phase blocking ELISA detection kit was purchased from Lanzhou Shouyan Biotechnology Co., Ltd. (Lanzhou, China). Mouse IL-4, IFN-γ, IgG1, and IgG2a ELISA kits were purchased from Jiangsu Meimian Industrial Co., Ltd. (Yancheng, China). Flow cytometry antibodies against mouse CD3-FITC, CD4-APC, and CD8-PE were purchased from BD Pharmingen (San Diego, CA, USA), and 4% paraformaldehyde, rabbit anti-MyD88/PI3K/AKT polyclonal antibody, and mouse anti-p65/p-p65/β-actin antibody were purchased from Shanghai Beyotime Biotechnology Co., Ltd. (Shanghai, China).

### 2.2. Vaccine Preparation

The FMD VLPs were produced in a prokaryotic system, where the FMDV capsid proteins (VP0, VP1, and VP3) were expressed as SUMO fusion proteins in *E. coli*. The SUMO tag was subsequently cleaved, leading to the formation of VLPs with size and morphology similar to that of the authentic FMDV. The WT was prepared as previously described [27] and consists of mineral oil (white oil), EL360, Tween 80, n-pentanol, and stearate-2. The VLPs were diluted in PBS (1 mM pH 7.4) to 100 μg/mL and then combined with ISA201 or WT in a 1:1 (*v*/*v*) ratio. The mixture was stirred at room temperature for 10 min at a low shear rate (300 r/min) to form a W/O/W emulsion.

### 2.3. Physical and Chemical Property Evaluation

The VLPs+WT was centrifuged at 3000 r/min for 15 min, and the volume of the water phase precipitated at the bottom was measured. The morphology was observed in bright-field by a confocal laser scanning microscopy (CLSM) (Zeiss LSM880, Jena, Germany). The dynamic viscosity of VLPs+WT at 25 °C was measured with a digital viscometer (Brookfield DV-II+Pro, Worcester, MA, USA) with the rotating cylinder method. The average particle size and polydispersity index (PDI) of VLPs+WT were determined via dynamic light scattering with a nanoparticle size analyzer (Zetasizer Nano ZSE, Malvern, UK).

### 2.4. Mice Immunization

BALB/c mice (4-week-old, female) were purchased from Yangzhou University Comparative Medicine Center. The mice were randomly divided into 4 groups, with 5 mice in each group. Each mouse was subcutaneously inoculated with 200 μL FMD vaccine (containing 10 μg FMD VLPs). The groups were as follows: (I) FMD VLPs (VLPs); (II) FMD VLPs+WT (VLPs+WT); (III) FMD VLPs+ISA201 (VLPs+201); (IV) Control (PBS). Serum samples were collected on days 14, 21, 28, 56, 70, and 84 post-immunization. All experimental procedures adhered to the guidelines of the Institutional Animal Care and Use Committee of Nanjing Agricultural University, ensuring compliance with animal welfare standards.

### 2.5. Safety Assay

To assess the biocompatibility of the VLPs+WT, the mice were weighed and monitored for behavior and local reactions on days 0, 7, 14, and 28 post-immunization. On day 14 post-immunization, the mice were euthanized, and major organs (heart, liver, spleen, lungs, kidneys) and the injection site skin were collected. These tissues were immersed in 4% paraformaldehyde, fixed and embedded in paraffin by standard method, sliced (4 mm), and stained with hematoxylin and eosin (H&E). The sections were observed under an optical microscope (Jiangnan XD-202, Nanjing, China).

### 2.6. Detection of Antibodies and Cytokines

Serum FMDV-specific antibody titers were determined by a liquid phase blocking (LPB) ELISA kit according to the manufacturer’s instructions: LPB-ELISA antibody titers ≥ 6 log2 (1:64) were considered to have protection. Briefly, serum samples were diluted in a 2-fold series using PBST in a U-shaped reaction plate, with 50 μL/well. The viral antigen was diluted to the working concentration and added at 50 μL/well to the serum and control wells. The plate was sealed and incubated at 37 °C for 90 min. The antigen–antibody mixture was then transferred to an ELISA plate coated with rabbit antibodies against FMD type O and incubated at 37 °C for 60 min. After washing with PBST, 50 μL of guinea pig antibody working solution was added per well, followed by a 30 min incubation at 37 °C. After further washing, 50 μL of rabbit anti-guinea pig IgG-HRP working solution was added and incubated for another 30 min at 37 °C. Following additional washes, TMB substrate solutions A and B were mixed in a 1:1 ratio and added to each well, incubating at 37 °C for 15 min. Finally, 50 μL of stop solution was added, and the optical density was measured at 450 nm. Additionally, commercial ELISA kits were used to determine antibody isotype (IgG1 and IgG2a), IFN-γ, and IL-4 levels in the serum on day 21. All experimental procedures were performed according to the protocols provided in the assay kit manuals.

### 2.7. Lymphoproliferation Assay

After 28 days of immunization, the spleens of mice were sterilely removed and placed on a cell strainer to grind into sterile PBS. The cell suspension was collected, centrifuged at 1200 r/min for 5 min, and the supernatant was discarded. To the cells, 1.8 mL of red blood cell lysis buffer was added, and they were lysed for 3 min and centrifuged at 1200 r/min for 5 min. The supernatant was then discarded. The cells were washed with sterile PBS and resuspended in RPMI 1640 medium containing 10% fetal bovine serum. After cell counting, the cells were placed in a 96-well plate at a concentration of 5 × 10^5^ cells/well. The final concentration of 10 μg/mL lipopolysaccharide (LPS) or 10 μg/mL FMD VLPs were added to the wells, with three replicates for each sample. After incubation at 37 °C, 5% CO_2_ for 24 h, 10 μL of CCK-8 reagent was added to each well and incubated for an additional 2 h. Absorbance at 450 nm was recorded by a microplate reader (Bio-Rad, Hercules, CA, USA). The stimulation index (SI) was calculated as:

$$SI=\frac{OD\;value\;of\;stimulated\;cells-OD\;value\;of\;culture\;medium}{OD\;value\;of\;the\;nonstimulated\;cells-OD\;value\;of\;culture\;medium}$$

### 2.8. Flow Cytometry Analysis

After 28 days of immunization, the mice spleens were sterilely taken, ground, and lysed. The spleen cells were washed with PBS, counted, and stained at a concentration of 1 × 10^6^ cells/150 µL. The T-cell surface was labeled with anti-mouse CD3-FITC, CD4-APC, and CD8-PE flow cytometry antibodies. The reaction was performed at 4 °C for 30 min in the dark, then washed twice with PBS. The expression levels of the above surface markers were examined by a flow cytometer (BD Accuri C6, San Jose, CA, USA). The proportions of CD3^+^CD4^+^ and CD3^+^CD8^+^ T lymphocytes were analyzed by FlowJo 10.8.1 software (BD Biosciences, Franklin Lakes, NJ, USA).

### 2.9. Western Blot

The spleen cells of each group were taken, and RIPA buffer containing protease inhibitors was added. The cells were incubated on ice for 30 min, centrifuged at 12,000 r/min for 15 min at 4 °C, and the supernatant was collected. Equal amounts of protein were mixed with sample buffer and boiled at 95 °C for 15 min. Proteins were separated on 12.5% polyacrylamide gel by SDS-PAGE and then transferred to PVDF membranes by wet transfer method at 100 V for 90 min. The membranes were blocked in 5% BSA at room temperature for 1 h. Membranes were then incubated overnight at 4 °C with primary antibodies against MyD88, PI3K, AKT, p65 and p-p65 (1:1000). After washing with TBST three times, membranes were incubated with secondary antibody (1:5000) anti-rabbit or -mouse IgG at room temperature for 1 h. Following three washes with TBST, enhanced chemiluminescence was added and the protein bands were detected on the imaging system (Bio-Rad ChemiDoc MP, Singapore).

### 2.10. Transcriptome Sequencing

On the 14th day post-immunization, spleens were collected from the VLP group and VLPs+WT group. Transcriptome sequencing was performed to detect gene expression changes in mouse spleens, with sequencing services provided by Biomarker Technologies Co., Ltd. (Beijing, China). During the detection of differentially expressed genes (DEGs), fold change ≥ 2 and FDR < 0.01 were used as criteria, and then GO and KEGG analysis were performed on the functions and pathways of DEGs.

### 2.11. Validation of Gene Expression by RT-qPCR

To validate the transcriptome sequencing results, 10 DEGs were detected with RT-qPCR. Specific primers were designed with Primerbank and synthesized by Beijing Tsingke Biotech Co., Ltd. (Beijing, China) (Table 1). The total RNA of the mouse spleen was reverse transcribed into cDNA, and then qPCR was conducted on a LightCycler 480 II (Roche Diagnostics, Basel, Switzerland). Each cDNA amplification was performed in triplicate, and Ct values were obtained for each sample. Relative quantification was performed using GAPDH mRNA expression as an internal reference. The amplification conditions were as follows: pre-denaturation at 95 °C for 2 min, denaturation at 95 °C for 15 s, annealing and extension at 60 °C for 30 s, for 40 cycles.

### 2.12. Statistical Analysis

Statistical analysis was performed using GraphPad Prism 9.0 software (San Diego, CA, USA). Data were presented as mean ± SEM. Unpaired *t*-test, one-way ANOVA, or two-way ANOVA analysis and Tukey’s multiple comparison test were used to analyze statistical differences between different groups. Statistical significance was defined as *p* values less than 0.05. * *p* < 0.05, ** *p* < 0.01, *** *p* < 0.001, and **** *p* < 0.0001.

## 3. Results

### 3.1. Characterization of VLPs+WT Vaccine

**Figure 2:**
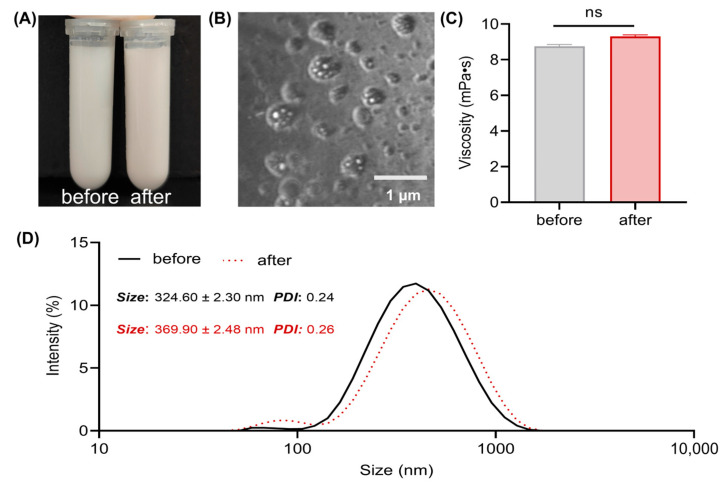
Physicochemical properties of VLPs+WT. Appearance (**A**), viscosity (**C**), particle size and PDI (**D**) of VLPs+WT before and after centrifugation for 15 min at 3000 r/min. (**B**) The morphology of VLPs+WT under CLSM. Scale bar = 1 µm. ns = no significant differences.

The VLPs+WT was a milky white emulsion and did not precipitate after centrifugation at 3000 r/min for 15 min (Figure 2A). The W/O/W morphology of the VLPs+WT can be observed under the CLSM (Figure 2B). The viscosities before and after centrifugation were 8.76 mPa·s and 9.30 mPa·s, respectively (Figure 2C). The VLPs+WT particle size was 324.60 ± 2.30 nm with a PDI of 0.24, and after centrifugation, the particle size was 369.90 ± 2.48 nm with a PDI of 0.26 (Figure 2D). Although the particle size increased slightly after centrifugation, the uniformity and viscosity remained satisfactory.

### 3.2. Safety Assessment

As described in Section 2.4, BALB/c mice were randomly divided into four groups (*n* = 5 per group) and subcutaneously inoculated with the FMD vaccine. Briefly, each mouse received 200 μL of the vaccine containing 10 μg of FMD VLPs. The changes of mental state, diet, drinking water, and body weight of mice after immunization can preliminarily evaluate the safety of VLPs+WT. Throughout the experiment, no abnormal behaviors or local reactions were observed. As shown in Table 2, the body weight of all mice in this study gradually increased.

**Figure 3:**
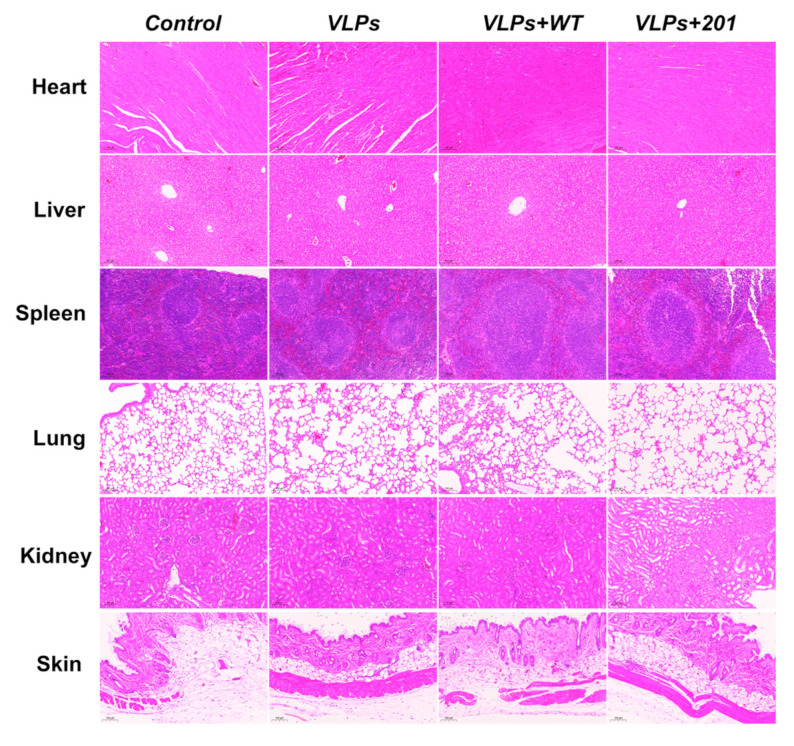
The biosafety of VLPs+WT in vivo. H&E-stained tissues of the vital organs (heart, liver, spleen, lung, kidney, skin) in BALB/c mice from different groups were examined at 14 days after immunization. Scale bar = 100 μm. Data are presented as the mean ± SEM, *n* = 3.

As shown in Figure 3, histological analysis indicated no oil residue, connective tissue proliferation, or damage in the skin at the injection site in mice. Additionally, compared to the control group, mice in the immunized groups showed no myocardial injury, no granular degeneration of hepatocytes, a clear structure of the central artery surrounding lymph nodules and marginal zones in the spleen white pulp, clear demarcation between the red and white pulp, no obvious abnormalities in the bronchial structure at all levels, clear alveolar wall structure, evenly distributed glomeruli, no significant abnormalities in the renal medulla, and no obvious hyperplasia in the renal interstitium.

### 3.3. Evaluation of IgG and Cytokine Responses

**Figure 4:**
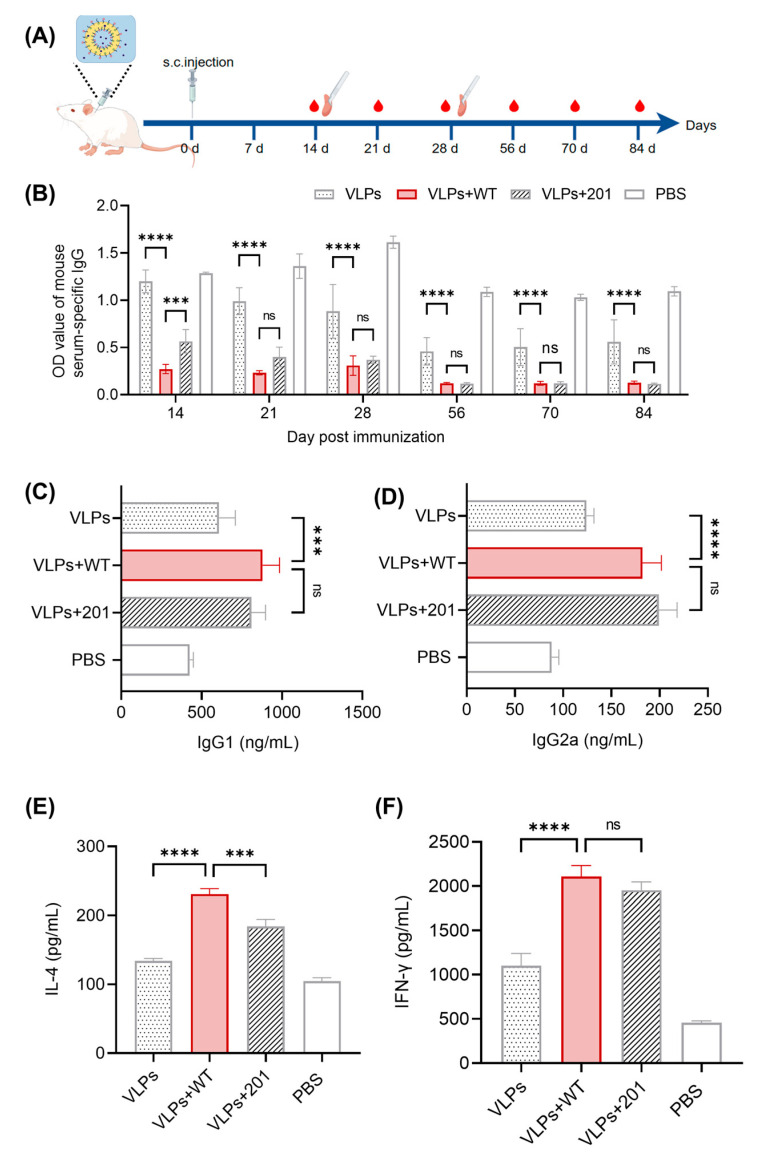
Experimental design, detection of specific antibodies, antibody subtypes, and cytokines. (**A**) Schematic illustration of the experimental process. s.c.: subcutaneous. (**B**) Specific IgG levels in serum were assessed using the FMD O-type antibody liquid-phase blocking ELISA on days 14, 21, 28, 56, 70, and 84 after vaccination. Data shown are endpoint titers at a dilution of 1:64. The concentration of IgG1 (**C**), IgG2a (**D**), IL-4 (**E**), and IFN-γ (**F**) in serum on day 21 after vaccination. Data were represented as mean ± SEM, *n* = 5. (ns = no significant differences; *** *p* < 0.001; **** *p* < 0.0001).

Antibody levels serve as a crucial indicator of vaccine efficacy [29]. The liquid-phase blocking ELISA is a widely adopted international method for assessing the immune effect of FMD, where higher OD values correspond to lower antibody levels. Immunization and blood collection were performed as shown in Figure 4A. From the level of induced specific antibody (Figure 4B), after 14 days of immunization, the VLPs+WT group demonstrated a significant difference compared to the VLP group (*p* < 0.0001) and the VLPs+201 group (*p* < 0.001). After 21 days of immunization, the WT was comparable to the ISA201 and continued to show a significant difference compared to the VLP group (*p* < 0.0001). The antibody level in the VLPs+WT group can last for nearly 3 months. ELISA results (Figure 4C–F) indicated that the VLPs+WT group had significantly higher levels of IgG1 (*p* < 0.001), IgG2a, IL-4, and IFN-γ (*p* < 0.0001) compared to the VLP group. Furthermore, the IL-4 level in the VLPs+WT group was also significantly higher than in the VLPs+201 group (*p* < 0.001). These results suggest that the VLPs+WT induces a robust Th2-dominated humoral immune response while also partially stimulating a cellular immune response.

### 3.4. Activation of Splenic T Cells

**Figure 5:**
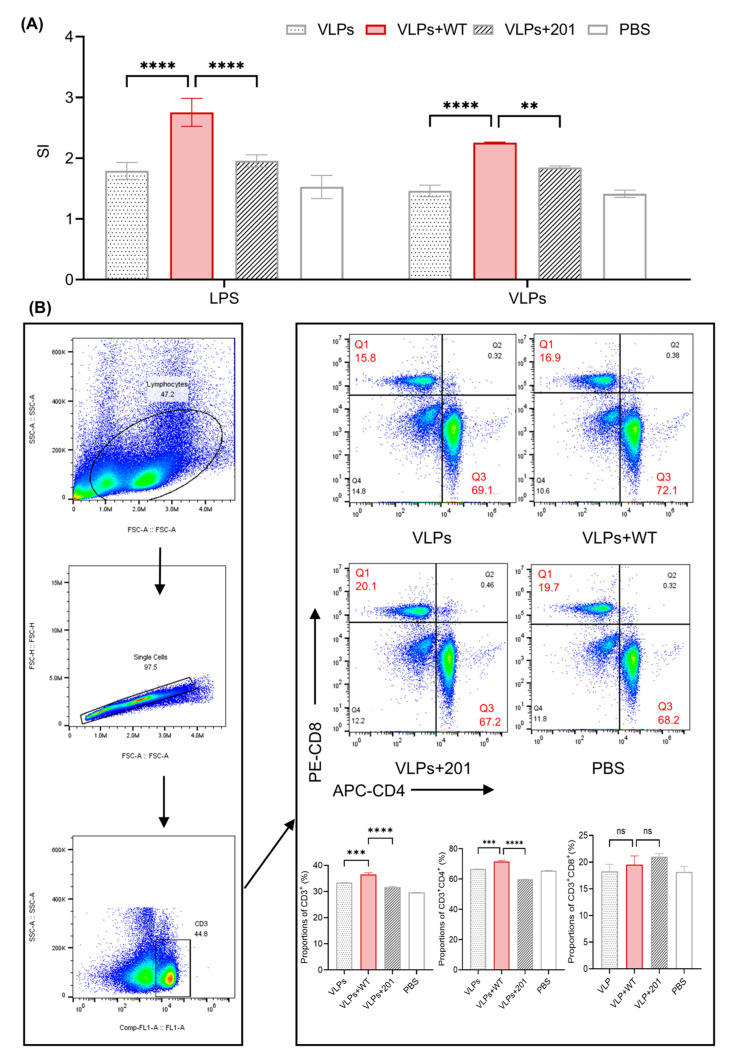
The proliferation and differentiation of spleen T lymphocytes. (**A**) Lymphocyte proliferation under LPS and VLPs stimulation were measured by CCK-8 assay. (**B**) Flow cytometry analysis of CD3^+^CD4^+^ and CD3^+^CD8^+^ T cells in the splenocytes after being immunized with PBS, VLPs, VLPs+WT, and VLPs+201. Data are shown as mean ± SEM, *n* = 3. (** *p* < 0.01, *** *p* < 0.001, **** *p* < 0.0001).

After 28 days of immunization, splenic lymphocytes were isolated from each group of mice, cultured in vitro, then stimulated with LPS and FMD VLPs antigens. As illustrated in Figure 5A, the lymphocyte proliferation results showed that under LPS stimulation, the SI value in the VLPs+WT group was 2.75, significantly higher than 1.79 in the VLPs group and 1.95 in the VLPs+201 group (*p* < 0.0001). When stimulated with FMD VLPs, the SI value in the VLPs+WT group was 2.25, significantly higher than 1.46 in the VLP group (*p* < 0.0001) and 1.85 in the VLPs+201 group (*p* < 0.01). These results indicated that the VLPs+WT stimulated lymphocyte proliferation.

Spleen cells were labeled and analyzed via flow cytometry to study T-cell differentiation. As illustrated in Figure 5B, the proportion of CD3^+^ T cells was 33.37% in the VLP group and significantly increased to 36.5% in the VLPs+WT group (*p* < 0.001), which aligns with the lymphocyte proliferation results. Furthermore, the proportion of CD3^+^CD4^+^ T cells was 66.6% in the VLP group and increased to 71.53% in the VLPs+WT group. This indicates that the WT adjuvant promotes a shift in T-cell differentiation towards a Th2-type response, supporting the activation of B cells and IgG antibody production. The CD3^+^CD8^+^ T-cell proportion was slightly reduced in the VLPs+WT group, which is likely due to the immune profile shift towards CD4^+^ T helper cells. This does not indicate an impaired immune response, but rather a redirection of the immune response, enhancing humoral immunity and antigen-specific antibody production.

### 3.5. Induction of PI3K-AKT and NF-κB Signaling Pathways

**Figure 6:**
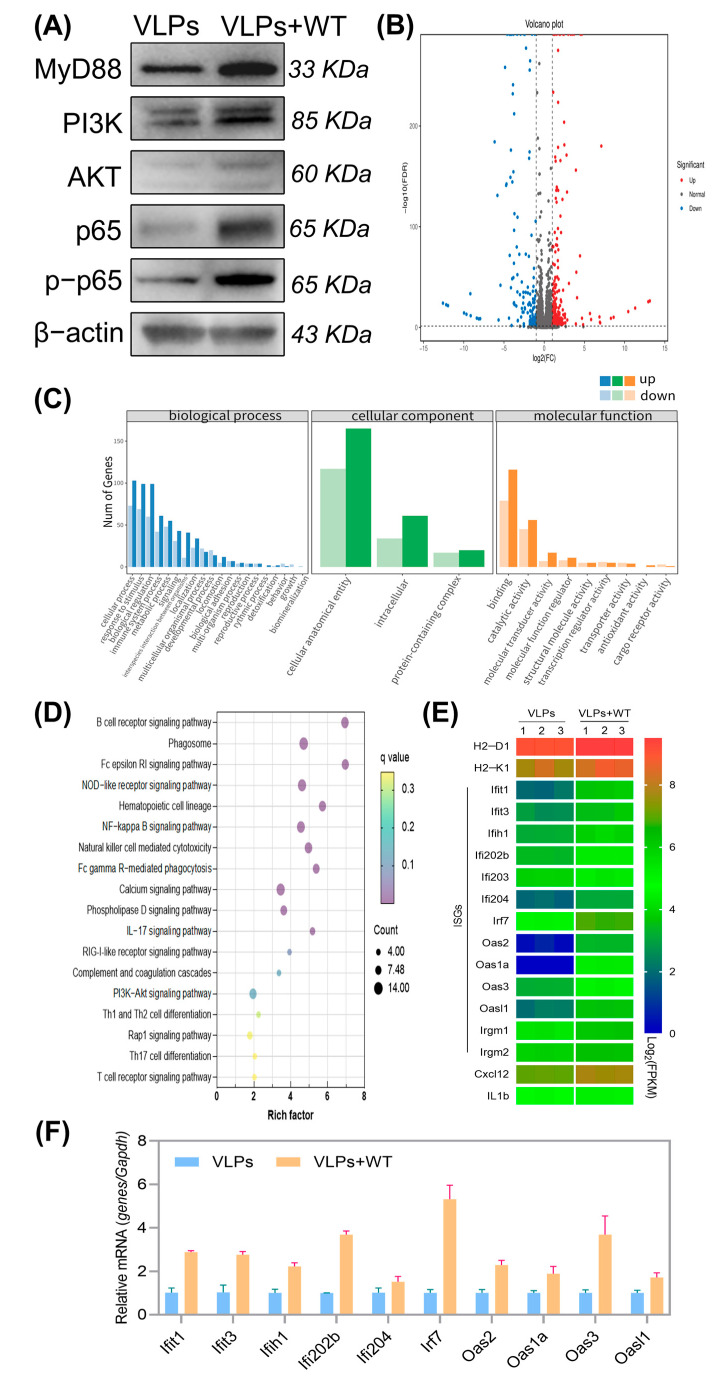
Western blot, the number of DEGs, enrichment analysis, and RT-qPCR results. (**A**) MyD88, PI3K, AKT, p65, p-p65 and β-actin protein bands were observed in the VLP group and VLPs+WT group. Mice (*n* = 3/group) were subcutaneously immunized with VLPs or VLPs+WT. Splenocytes were isolated from the spleens harvested 2 weeks after immunization and used for transcriptome sequencing. (**B**) The volcano plot shows the numbers of up-regulated and down-regulated genes. (**C**) GO functional enrichment analysis. GO terms with fold change ≥ 2 and FDR < 0.01 were considered significantly enriched by DEGs. (**D**) Enrichment of KEGG pathways analysis. The *x*-axis means the value of the gene ratio and the *y*-axis means the enrichment term of the pathway. The size of the dot on behalf of the number of DEGs and the color represents q values. (**E**) Heatmap of some DEGs in spleen cells. The color key indicates reads per kilobase per million reads normalized log2 transformed counts. (**F**) Validation of DEGs with RT-qPCR. Data were normalized to the expression of GAPDH, *n* = 3.

To further investigate whether the WT can activate the PI3K-AKT and NF-κB signaling pathways, we extracted proteins from the spleens of the mice and detected the expression of key proteins in these two pathways. In the VLPs+WT group, the levels of MyD88, PI3K, AKT, p65, and p-p65 proteins were increased (Figure 6A), indicating the upregulation of these signaling pathways. The active PI3K-AKT signaling pathway suggested that the WT stimulated stronger cell proliferation or survival. The increased levels of NF-κB signaling pathway proteins (p65 and p-p65) and MyD88 suggested that the WT activated a stronger immune response.

### 3.6. Differentially Expressed Genes (DEGs) and Functional Enrichment Analysis

In this study, we analyzed the gene expression of spleen cells from mice immunized with FMD VLPs and VLPs+WT by RNA sequencing. As shown in Figure 6B, compared to the VLP group, there were 443 DEGs in the VLPs+WT group, with 246 genes upregulated and 197 genes downregulated. The GO enrichment analysis of DEGs includes three main categories: biological process, cellular components, and molecular function. The function of DEGs was analyzed by GO database. As shown in Figure 6C, compared to the VLP group, the VLPs+WT group exhibited high expression in biological processes such as “cellular process”, “response to stimulus”, and “biological regulation” indicating that the WT promotes the interaction between the antigen and immune cells. In the cellular component category, “cellular anatomical entity”, was highly expressed, while in the molecular function category, “binding” was highly expressed.

KEGG is a database containing pathway maps that represent molecular interaction and reaction networks. As shown in Figure 6D, compared to the VLP group, the VLPs+WT group significantly regulated the “B-cell receptor signaling pathway” and “Phagosome”. This corresponds with the Western blot results, where PI3K is identified as an important downstream effector in the B-cell receptor signaling pathway. KEGG analysis results also confirmed that the WT activated the NF-κB signaling pathway and the PI3K-AKT signaling pathway. Figure 6E shows that major histocompatibility complex (*MHC*) proteins (*H2-D1* and *H2-K1*) were significantly upregulated in the VLPs+WT group. Additionally, there was a significant upregulation of interferon-stimulated genes (ISGs: *Ifit1*, *Ifit3*, *Ifih1*, *Irf7*, *Oas3*, etc.). The Chemokine Cxcl12 can enhance the recruitment of immune cells to the injection site.

In order to verify the results of transcriptome sequencing, we selected the 10 DEGs in Figure 6F and measured gene expression with RT-qPCR. The data from RT-qPCR were highly correlated with the data from transcriptome sequencing (Figure 6E), indicating the reliability of the transcriptome sequencing results.

## 4. Discussion

In this study, we investigated the immunogenicity and safety profile of VLPs combined with WT as an adjuvant in a mouse model. The formulation resulted in a stable milky white W/O/W emulsion. Compared to the W/O formulation, the W/O/W formulation has lower irritancy and induces a stronger immune response than the O/W formulation [30,31]. The observed increase in particle size post-centrifugation, although slight, did not significantly compromise the uniformity or viscosity. According to Ostwald’s maturation theory, the main reason for the instability of the emulsion is the fusion of droplets [32]. The smaller droplet diameter helps to avoid Ostwald’s maturation, which is conducive to the preservation of the vaccine. In addition, studies have shown that the larger the particle size, the lower the lymph node transmission efficiency and the ability to target dendritic cells [33,34]. Oil-based emulsion adjuvants create an “immunocompetent environment” at the injection site [19], with side effects mainly including ulceration or granuloma [35]. Safety assessment indicated that there were no abnormal behavioral changes or local reactions in mice. Histological analysis revealed no oil residue or tissue damage, demonstrating the good biocompatibility of the VLPs+WT vaccine.

The efficacy of a vaccine is primarily assessed by the levels of antibodies it induces. Countries free from FMD rely on high-potency “emergency” vaccines to control outbreaks, necessitating the development of vaccines capable of rapidly eliciting immune responses for effective protection [25]. Our experimental results indicate that the VLPs+WT group exhibited significantly elevated antibody levels at 14 days post-immunization compared to both the VLP and VLPs+201 groups, highlighting its advantages as an “emergency” vaccine. Moreover, the antibody levels in the VLPs+WT group were sustained for 12 weeks, suggesting the WT promoted long-term interactions between antigens and immune cells, resulting in a “depot effect” [36]. Although the VLPs+WT vaccine demonstrated enhanced immune responses compared to the VLPs alone, the increase in immunogenicity relative to the ISA201 adjuvant vaccine was moderate. Antibody subtypes play an important role in preventing FMD [37]. The production of IgG1 is associated with the Th2-mediated humoral immune response, while IgG2a is Th1-mediated and is mainly found in cell-mediated immune responses [38,39]. The VLPs+WT group not only produced higher levels of IgG1, indicative of a Th2-dominated humoral response, but also showed increased levels of IL-4 and IFN-γ. The elevation of IL-4 is particularly noteworthy, as it plays a crucial role in B-cell proliferation and activation, facilitating enhanced IgG1 production [40]. The presence of IgG2a and Th1 cytokine IFN-γ suggests that the VLPs+WT vaccine may also engage cellular immune responses. Natural host immunity to the FMD virus is potentially T-cell dependent [38]. Our flow cytometry analysis revealed a significant increase in the proportion of CD3^+^ T cells from 33.37% in the VLP group to 36.5% in the VLPs+WT group, which corresponds with the observed increase in lymphocyte proliferation. Interestingly, the proportion of CD3^+^CD4^+^ T cells in the VLP and VLPs+201 groups was lower than in the PBS group. This decrease may be attributed to the differentiation of a larger proportion of CD3^+^ T cells into CD3^+^CD8^+^ T cells, which are crucial for cellular immunity and viral clearance. In contrast, the proportion of CD3^+^CD4^+^ T cells significantly increased in the VLPs+WT group, rising from 66.6% to 71.53%. This suggests that the WT adjuvant promotes a shift towards a Th2-dominant immune response, which is important for enhancing B-cell activation and stimulating the production of IgG antibodies. However, while the immune responses observed in this study are promising, the absence of protection data remains a limitation. Future research should include protection assays to further evaluate the vaccine’s ability to confer immunity against FMD, particularly in larger-animal models such as pigs. This will provide a more comprehensive understanding of the vaccine’s potential in real-world applications.

Transcriptome analysis based on RNA sequencing is a tool for exploring complex physiological pathways, including immune responses. However, only a few studies have utilized transcriptomes to analyze the effects of vaccine adjuvants. For instance, Yuan’s group employed transcriptome analysis to evaluate the adjuvant effect of ginsenoside-containing sunflower seed oil on the immune response induced by inactivated Newcastle disease virus in chickens [41]. In this study, we conducted a comprehensive analysis of gene expression in the spleen cells of mice immunized with FMD VLPs and VLPs+WT vaccine. Compared to the VLP group, RNA sequencing in the VLPs+WT group identified 443 DEGs. GO enrichment analysis revealed significant upregulation of “cellular processes”, “response to stimulus”, “biological regulation”, and “binding”, suggesting that the WT adjuvant enhanced interactions between VLPs and immune cells, which is critical for an effective immune response. Our KEGG pathway analysis indicated that the VLPs+WT vaccine notably influenced the “B-cell receptor signaling pathway” and “Phagosome”. B cells recognize antigens via the B-cell receptor (BCR) on their surface, which, upon stimulation, drives activation and differentiation into antibody-producing plasma cells [42]. One of the main signaling pathways mediated by BCRs is the PI3K-AKT pathway, consistent with our Western blot results. Activation of the PI3K-AKT pathway is crucial for mediating cell growth, proliferation, and survival, as well as balancing Th1 and Th2 immune responses [43]. In summary, the WT adjuvant significantly boosted the immune response to FMD VLPs by modulating gene expression associated with key immune pathways.

## 5. Conclusions

In this study, we prepared a W/O/W adjuvant (named WT) combined with an FMD VLP antigen, which demonstrated low viscosity (8.76 mPa·s), a particle size of 324.60 ± 2.30 nm, and good stability and safety. This combination significantly enhanced specific antibody levels; increased lymphocyte proliferation, particularly improving humoral immune responses; and activated the B-cell receptor signaling and phagosome pathways. Overall, the VLPs+WT provided a better immune response in mice, offering a theoretical basis for further expanding its application in host animals.

## Figures and Tables

**Table 1:** Sequences of primers for RT-qPCR.

Gene	Primer Sequence (5′ to 3′)
*Ifit1*	**F:** CTGAGATGTCACTTCACATGGAA
	**R:** GTGCATCCCCAATGGGTTCT
*Ifit3*	**F:** CCTACATAAAGCACCTAGATGGC
	**R:** ATGTGATAGTAGATCCAGGCGT
*Ifih1*	**F:** ACTTGCTTCGAGAAGGGACTA
	**R:** AGCTCTCTTACACCTGACTCATT
*Ifi202b*	**F:** GACCCCTTCCAGTGATTCATCT
	**R:** ACAGCACCTTTGCTAATGTTCT
*Ifi204*	**F:** GACAACCAAGAGCAATACACCA
	**R:** ATCAGTTTGCCCAATCCAGAAT
*Irf7*	**F:** GAGACTGGCTATTGGGGGAG
	**R:** GACCGAAATGCTTCCAGGG
*Oas2*	**F:** TTGAAGAGGAATACATGCGGAAG
	**R:** GGGTCTGCATTACTGGCACTT
*Oas1a*	**F:** GCCTGATCCCAGAATCTATGC
	**R:** GAGCAACTCTAGGGCGTACTG
*Oas3*	**F:** TCTGGGGTCGCTAAACATCAC
	**R:** GATGACGAGTTCGACATCGGT
*Oasl1*	**F:** CAGGAGCTGTACGGCTTCC
	**R:** CCTACCTTGAGTACCTTGAGCAC
*Gapdh*	**F:** AGGTCGGTGTGAACGGATTTG
	**R:** TGTAGACCATGTAGTTGAGGTCA

**F:** forward primer; **R:** reverse primer.

**Table 2:** Bodyweight changes in mice (mean ± SEM, gram, *n* = 5).

Day Post-Immunization	PBS	VLPs	VLPs+WT	VLPs+201
0	21.49 ± 0.26	20.94 ± 0.27	21.18 ± 0.18	21.11 ± 0.18
7	22.20 ± 0.21	22.29 ± 0.23	22.42 ± 0.19	22.25 ± 0.24
14	22.81 ± 0.16	22.94 ± 0.20	23.08 ± 0.19	23.03 ± 0.12
21	23.33 ± 0.25	23.40 ± 0.21	23.58 ± 0.27	23.48 ± 0.08
28	23.62 ± 0.10	23.81 ± 0.28	23.88 ± 0.20	24.00 ± 0.23

## Data Availability

The raw data supporting the conclusions of this article will be made available by the authors on request.
